# Gray-white matter covarying impairment in the anterior cingulate cortex revealed by multimodal meta-analysis of post-stroke cognitive impairment

**DOI:** 10.3389/fnagi.2026.1654758

**Published:** 2026-02-23

**Authors:** Zimeng Tang, Ruonan Huang, Xin Tan, Longjian Huang

**Affiliations:** 1 Youjiang Medical University for Nationalities, Baise, Guangxi, China; 2 Department of Physical Education, Yulin University, Yulin, Shaanxi, China

**Keywords:** anterior cingulate cortex, gray matter atrophy, multimodal meta-analysis, post-stroke cognitive impairment, PSCI, white matter microstructure

## Abstract

**Background:**

Post-stroke cognitive impairment (PSCI) involves complex interactions between gray matter (GM) and white matter (WM) pathology, yet their covarying patterns remain poorly characterized.

**Methods:**

We conducted a multimodal meta-analysis following PRISMA guidelines (PROSPERO registration number: CRD420251078162). Systematic literature searches identified 7 gray matter volume (GMV) (292 PSCI patients/231 controls) and 8 diffusion tensor imaging fractional anisotropy (DTI-FA) studies (339 PSCI patients/258 controls). Seed-based d Mapping with Permutation of Subject Images (SDM-PSI) was used for voxel-wise meta-analyses. Spatial integration identified GM-WM covarying pathology.

**Results:**

PSCI patients showed significant global GMV reduction versus controls (*p* = 0.011). Family-wise error (FWE)-corrected analyses revealed GM atrophy in the right medial superior frontal gyrus (BA 10), right superior temporal gyrus/temporal pole (BA 38), and left orbital middle frontal gyrus (BA 47) (all *p* < 0.05, FWE-corrected). Concurrent GMV reduction and FA decrease occurred in the left anterior cingulate/paracingulate gyri (BA 11/25; *p* = 0.043, uncorrected). Meta-regression demonstrated negative associations between age and GMV in the right medial superior frontal gyrus (BA 10) (*p* = 0.006) and FA in the left median network, cingulum (*p* = 0.021). Heterogeneity was low (*I*^2^ < 50%). Egger’s test suggested potential publication bias for the right temporal pole and DTI findings.

**Conclusion:**

This study applied SDM-PSI multimodal meta-analysis to delineate characteristic neurostructural damage patterns in PSCI. Key findings encompass gray matter atrophy within frontotemporal cognitive hubs (FWE-corrected). Additionally, exploratory analyses suggested potential covarying GM-WM pathology in the left anterior cingulate/paracingulate gyri (BA 11/25; *p* = 0.043, uncorrected). These findings elucidate PSCI pathogenesis and suggest potential anatomical targets for future research.

## 1 Introduction

Cerebrovascular diseases represent a significant global public health burden, constituting the second most prevalent cause of mortality worldwide. Epidemiological studies demonstrate that 30–40% of stroke survivors develop post-stroke cognitive impairment (PSCI). This condition is characterized by multidomain cognitive dysfunction, particularly affecting memory and executive functions, which significantly impairs patients’ quality of life and adversely impacts rehabilitation outcomes (Attanasio et al., 2024; Huang et al., 2022; Pendlebury and Rothwell, 2019). Nevertheless, the current understanding of PSCI neuropathological mechanisms remains incomplete, significantly impeding the development of targeted therapies for cognitive rehabilitation. This knowledge gap positions PSCI as a crucial unresolved challenge in secondary stroke prevention. Although previous unimodal neuroimaging investigations have identified localized gray matter atrophy (Hobden et al., 2023; Zhang et al., 2025) and white matter microstructural (Coenen et al., 2024; Seyman et al., 2023) alterations in PSCI, the complex interplay between GM and WM, including potential synergistic or competitive interactions, remains poorly understood. Most critically, the bidirectional relationship between post-stroke neuroplastic compensatory mechanisms and progressive white matter degeneration—and their collective impact on cognitive trajectory—remains to be systematically investigated (Gallucci et al., 2024; Khan et al., 2025).

To bridge this gap, we employed SDM-PSI (Seed-based d Mapping with Permutation of Subject Images) multimodal fusion analysis to quantify structural covariance patterns using voxel-wise joint probability mapping (Albajes-Eizagirre et al., 2019). Our approach aimed to integrate gray matter nuclei degeneration with white matter pathway disruption, providing insights into PSCI-related neural circuit pathophysiology. This study utilized voxel-wise, multimodal meta-analysis using Seed-based d Mapping with Permutation of Subject Images (SDM-PSI) to characterize concurrent gray matter volume (GMV) (Lee et al., 2022) and white matter microstructural [DTI fractional anisotropy (FA)] (Kimura et al., 2020) abnormalities in PSCI. Through spatial integration of GM atrophy and WM integrity measures, we identified critical hubs of GM-WM covarying pathology. To elucidate preclinical neurodegenerative processes, we conducted meta-regression analyses to quantify relationships between age, cognitive performance metrics, and neurostructural alterations in PSCI. Guided by existing neurobiological evidence, we hypothesized region-specific structural-functional decoupling in PSCI patients, with the meta-analytic approach designed to establish robust, reproducible neural signatures of PSCI-related neurodegeneration.

## 2 Materials and methods

### 2.1 Literature search

This study was conducted in strict accordance with the Preferred Reporting Items for Systematic Reviews and Meta-Analyses (PRISMA) guidelines and prospectively registered in the PROSPERO international prospective register of systematic reviews (Registration: CRD420251078162) (Page et al., 2021). To systematically examine structural covariance patterns between gray matter volume (GMV) and white matter integrity (assessed via diffusion tensor imaging fractional anisotropy [DTI-FA]) in PSCI, we implemented dual systematic search strategies targeting GMV and DTI-FA studies independently. Our comprehensive literature searches spanned three major databases (PubMed, Embase, and Web of Science) for publications through May 15, 2025. The GMV search strategy incorporated the following Medical Subject Headings (MeSH) terms: “Stroke”[Mesh], “Cognitive Dysfunction”[Mesh], and “Gray Matter”[Mesh], while the DTI search utilized “Stroke”[Mesh], “Cognitive Dysfunction”[Mesh], and “Diffusion Tensor Imaging”[Mesh] (complete search syntax for all databases is detailed in Supplementary Tables 1–3).

### 2.2 Study selection

Studies had to meet the following inclusion criteria: (1) whole-brain level neuroimaging analyses comparing gray matter (reflected by brain structural size) and white matter (represented by FA values) among post-stroke cognitive impairment patients, healthy controls (HC), and post-stroke patients without cognitive impairment; (2) original articles published in peer-reviewed English or Chinese journals; (3) reporting whole-brain changes using standardized three-dimensional stereotaxic coordinates [Montreal Neurological Institute (MNI) space].

Studies were excluded based on the following criteria: (1) cases involving trauma/invasive procedures, hemorrhagic stroke, or pre-stroke dementia/cognitive impairment diagnoses; (2) review articles, case reports, dissertations, etc.; (3) publications in languages other than English or Chinese; (4) animal studies; (5) research focusing on specific ROIs or reporting only ROI-based coordinates; (6) studies with data overlapping other publications.

### 2.3 Quality assessment and data extraction

#### 2.3.1 Basic data

The following descriptive information was extracted from each included study: authors, sample size, age, Mini-Mental State Examination (MMSE) scores (Wei et al., 2025), whole-brain gray matter volume values, fractional anisotropy (FA), full width at half maximum (FWHM), voxel size, statistical significance thresholds for primary analyses, stereotaxic coordinate data, as well as peak coordinates and their corresponding statistical heights (Zhu et al., 2022). Of particular note, the peak values could be expressed in various statistical formats (*p*-values, *t*-values, or *z*-values), which were subsequently converted to standardized z-scores using the “Convert peaks” module in the SDM-PSI GUI software for unified statistical analysis.

#### 2.3.2 Quality assessment

Prior to conducting the meta-analysis, the quality of included studies was systematically evaluated using a 10-item checklist (Supplementary Table 4; Zhong et al., 2023), which assessed three key domains: (1) participant characteristics (items 1–4), (2) neuroimaging methodology and analytical approaches (items 5–8), and (3) results interpretation and conclusions (items 9–10). This comprehensive evaluation examined clinical and demographic characteristics of study samples as well as neuroimaging protocols. Two investigators independently performed literature screening, study selection, and quality assessment. Any discrepancies between reviewers were resolved through consultation with a third senior researcher. Ultimately, all studies meeting our predefined quality threshold (score > 6.0) were included in the final analysis (Supplementary Tables 5, 6).

### 2.4 Data analysis

#### 2.4.1 Analysis of whole-brain gray matter volume

Prior to performing voxel-wise meta-analyses, we conducted a comprehensive GMV assessment. This preliminary analysis systematically quantified whole-brain GM alterations using the “Globals” utility within SDM-PSI. We extracted and standardized mean global GM values and their corresponding standard deviations from all eligible studies. This module calculated the pooled effect size (Hedges’ g) (Lee et al., 2015) to assess the magnitude of global atrophy, while simultaneously evaluating between-study heterogeneity through the I^2^ statistic (Crippa et al., 2016).

#### 2.4.2 A meta-analysis was performed on both gray matter and white matter alterations

To integrate analyses of GMV and white matter DTI findings, we employed Seed-based d Mapping with Permutation of Subject Images (SDM-PSI, version 6.23) for separate meta-analyses. SDM-PSI represents an advanced neuroimaging meta-analytic tool that utilizes Permutation of Subject Images (PSI) technology to evaluate whether brain statistical map effects are significantly non-zero (Suchting et al., 2021). Multiple studies have employed the Seed-based d Mapping with Permutation of Subject Images (SDM-PSI) method for meta-analyses to identify alterations in brain structure and function across various disorders and conditions (Kong et al., 2024; Nan et al., 2024; Zhang et al., 2021; Zhang et al., 2023). This approach surpasses traditional binary *p*-value classification, enabling more nuanced graded assessment of evidence strength. The methodology synthesizes all relevant information into a unified brain map by incorporating peak coordinates and their corresponding statistical values (*t*-scores) reported in individual studies (Radua et al., 2012). These analyses were implemented following the official SDM-PSI tutorial^1^ and the accompanying software manual. Resultant SDM maps were visualized using MRIcron software^2^. All procedures strictly adhered to the protocol established by Radua et al. (2012), employing default SDM core parameters (full width anisotropy = 1, isotropic full-width at half maximum [FWHM] = 20 mm, voxel size = 2 mm) to optimize the balance between Type I and Type II errors (Radua et al., 2014) (Radua et al., 2014). To mitigate Type I error risk (false rejection of true null hypotheses) from multiple comparisons while maintaining sensitivity, results underwent family-wise error (FWE) correction at the cluster level (*p* < 0.05, with a minimum cluster size threshold = 10 voxels) (McHugh, 2011).

#### 2.4.3 Covariance analysis of gray matter and white matter alterations

To systematically investigate the spatial convergence between gray matter and white matter abnormalities, we performed a multimodal fusion analysis (Tur et al., 2022) using the dedicated “Multimodal” processing module embedded within SDM-PSI (version 6.23). This approach facilitates the voxel-wise assessment of pathological overlap across distinct imaging modalities by performing a conjunction analysis of the meta-analytic maps. Specifically, the corrected effect size maps derived from the independent GMV and DTI meta-analyses (Ghaderi et al., 2024) were utilized as inputs. The software computed spatial convergence by assessing the union of null hypotheses. While we prioritized stringent FWE correction for robust inference, no overlapping regions survived at this level. Therefore, to facilitate an exploratory analysis of potential structural abnormalities and mitigate the risk of Type II errors (false negatives), we applied a significance threshold of uncorrected *p* < 0.05, combined with a minimum cluster extent threshold of 10 voxels.

#### 2.4.4 Analyses of heterogeneity and publication bias

To assess result robustness, we quantified heterogeneity using the I^2^ statistic, with thresholds defined as follows: low heterogeneity (*I*^2^ < 50%), moderate heterogeneity (50% ≤ *I*^2^ ≤ 75%) (Egger et al., 1997). Publication bias was evaluated through dual approaches: (1) visual inspection of funnel plot symmetry, and (2) Egger’s linear regression test. Significant publication bias was indicated by concurrent presence of both funnel plot asymmetry and statistically significant Egger’s test results (*p* < 0.1) (Furuya-Kanamori et al., 2018).

#### 2.4.5 Meta-regression analyses

To evaluate potential influences of demographic variables (e.g., age) and cognitive measures (MMSE scores), we performed meta-regression analyses. In accordance with SDM software recommendations, these analyses employed a more conservative significance threshold (uncorrected *p* < 0.005, SDM-PSI default setting) to minimize false positive findings. Results failing to meet this stringent threshold (i.e., those beyond the scope of primary meta-analyses) were not reported (Su et al., 2021).

## 3 Results

### 3.1 Included studies and sample characteristics

**FIGURE 1:**
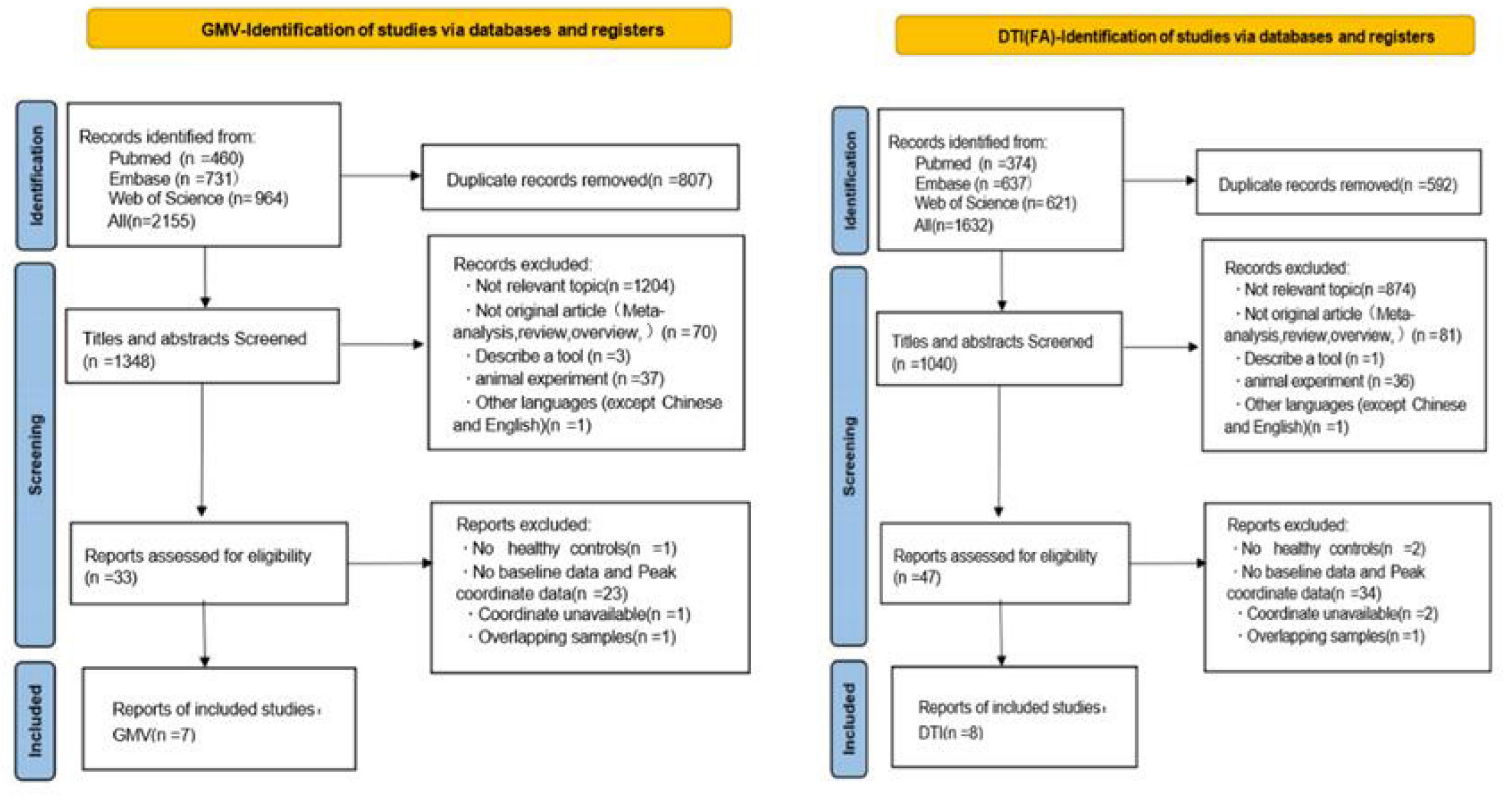
Flowchart to identify eligible studies for the meta-analysis. VBM, voxel-based morphometry; DTI, diffusion tensor imaging.

Figure 1 presents the PRISMA flow diagram detailing the systematic literature search and study selection process. For gray matter analyses, we included 7 GMV imaging studies comprising 292 post-stroke cognitive impairment patients and 231 matched controls. Regarding white matter investigations, 8 DTI studies were incorporated, encompassing 339 PSCI patients and 258 matched controls (Table 1). Subjects lacking complete age or gender information were excluded from statistical analyses. Comprehensive summaries of demographic characteristics, clinical profiles, neuroimaging features, and quality assessment scores for all included studies are provided in Supplementary Tables 5, 6.

**TABLE 1:** Demographic information of meta-analysis samples.

Characteristic	PSCI	control group	*P*-value
**GMV**			
Sample, n	292	231	–
Mean age, y	54.13 ± 18.97	55.92 ± 13.38	0.326
Male/Female, n	140/152	126/105	0.248
MMSE	25.51 ± 3.25	28.63 ± 1.15	< 0.001
**DTI**			
Sample, n	339	258	–
Mean age, y	54.60 ± 9.18	55.40 ± 10.74	0.375
Male/Female, n	189/150	143/115	0.930
MMSE	25.95 ± 4.17	28.28 ± 3.80	< 0.001

PSCI, post-stroke cognitive impairment; GMV, gray matter volume; DTI, diffusion tensor imaging.

### 3.2 Total brain gray matter volume

Our integrated meta-analysis of whole-brain gray matter volume (GMV) measurements demonstrated significant volumetric reductions in post-stroke cognitive impairment (PSCI) patients relative to healthy controls (Supplementary Table 7). Random-effects modeling yielded a standardized mean difference of Hedges’ g = −0.647 (95% CI: −1.148 to −0.146), representing a moderate effect size that was statistically robust (*z* = -2.531, *p* = 0.011). This finding indicates consistent gray matter atrophy across studies, with PSCI patients showing approximately 0.65 SD units lower GMV than matched controls.

### 3.3 GMV meta-analysis

**FIGURE 2:**
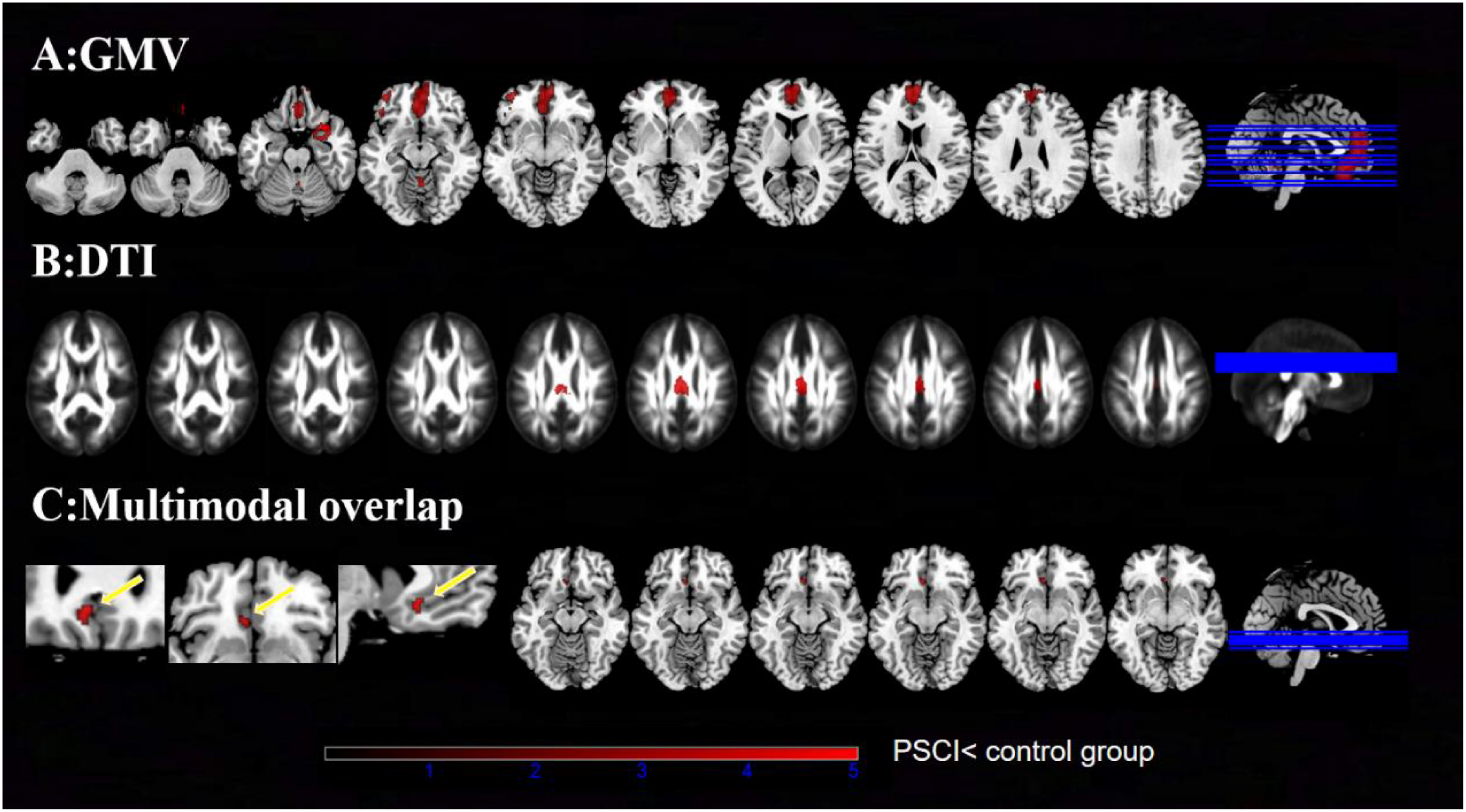
Meta-analyses results regarding **(A)** GMV difference between PSCI and control group. **(B)** Meta-analyses results regarding DTI-FA difference between PSCI and control group. **(C)** Overlap of GMV and DTI alterations. The left panels show enlarged views (sagittal, coronal, axial) of the anterior cingulate cortex (ACC), where concurrent gray matter atrophy and FA decrease are indicated by yellow arrows. The right panels display the corresponding whole-brain slices. PSCI, post-stroke cognitive impairment; GMV, gray matter volume; DTI, diffusion tensor imaging. FA, fractional anisotropy.

The SDM-PSI-based meta-analysis of gray matter volume (GMV) revealed widespread cerebral atrophy in post-stroke cognitive impairment (PSCI) patients compared to matched controls, as illustrated in Figure 2A and Table 2. Following stringent family-wise error (FWE) correction (*p* < 0.05), the atrophy pattern converged to three robust regions: (1) the right medial superior frontal gyrus (BA 10, exhibiting the most extensive atrophy), (2) the right temporal pole (BA 38), and (3) left orbital middle frontal gyrus (BA 47).

**TABLE 2:** Meta-analyses results regarding GMV difference between PSCI and control group.

Local maximum	Peak MNI coordinate (x, y, z)	SDM-Z value	*p*-value	No. of voxels	Egger’s test (*p*-value)	*I* ^2^	Cluster breakdown	
							Brain Region	No. of voxels
***p* < 0.05 TFCE-corrected**								
Right superior frontal gyrus, medial, BA 10	4, 58, 14	−4.946	< 0.001	2,440	0.988	2.78 (*I*^2^< 50%)	Right superior frontal gyrus, medial, BA 10	307
							Left superior frontal gyrus, medial, BA 10	263
							Right superior frontal gyrus, medial orbital, BA 11	203
							Right gyrus rectus, BA 11	186
							Left superior frontal gyrus, medial orbital, BA 11	164
							Left gyrus rectus, BA 11	159
							Corpus callosum	118
							Left superior frontal gyrus, medial, BA 32	110
							Left anterior cingulate/paracingulate gyri, BA 10	107
							Right superior frontal gyrus, medial orbital, BA 10	91
							Right anterior cingulate/paracingulate gyri, BA 32	90
							Left anterior cingulate/paracingulate gyri, BA 32	88
							Left superior frontal gyrus, medial orbital, BA 10	73
							Left superior frontal gyrus, medial	67
							Left anterior cingulate/paracingulate gyri, BA 11	55
							Right anterior cingulate/paracingulate gyri, BA 10	35
							Right superior frontal gyrus, medial orbital	34
							Left anterior cingulate/paracingulate gyri	32
							Left superior frontal gyrus, medial orbital	32
							Right superior frontal gyrus, orbital part, BA 11	24
							Right superior frontal gyrus, medial, BA 32	19
							Right superior frontal gyrus,medial	18
							Left gyrus rectus	15
							Right anterior cingulate/paracingulate gyri, BA 11	13
							Right striatum	8
							Right anterior cingulate/paracingulate gyri	7
							Right gyrus rectus	7
							Right superior frontal gyrus, medial, BA 9	5
							Right superior frontal gyrus, dorsolateral, BA 10	2
							Right superior frontal gyrus, dorsolateral, BA 9	1
							Right frontal orbito-polar tract	1
Right temporal pole, superior temporal gyrus, BA 38	28, 12, −26	−5.409	0.003	369	*P* < 0.001	6.79 (*I*^2^< 50%)	Right temporal pole, superior temporal gyrus, BA 38	84
							Right parahippocampal gyrus, BA 28	41
							Right amygdala, BA 34	34
							Right inferior frontal gyrus, orbital part, BA 38	21
							Right temporal pole, superior temporal gyrus	18
							Right parahippocampal gyrus, BA 38	16
							Right insula, BA 38	15
							Right inferior frontal gyrus, orbital part	15
							Right insula	11
							Right amygdala	32
							Right parahippocampal gyrus, BA 34	11
							Right temporal pole, superior temporal gyrus, BA 20	7
							Right temporal pole, superior temporal gyrus, BA 34	3
							Right insula	3
							Right temporal pole, superior temporal gyrus, BA 28	3
							Right inferior frontal gyrus, orbital part, BA 47	2
							Right parahippocampal gyrus, BA 28	2
							Right olfactory cortex, BA 34	2
							Right amygdala	2
							Right olfactory cortex	1
							Right parahippocampal gyrus, BA 36	1
							Right parahippocampal gyrus	1
Left middle frontal gyrus, orbital part, BA 47	−38, 50, −8	−4.658	0.018	113	0.994	1.55 (*I*^2^< 50%)	Left middle frontal gyrus, orbital part, BA 47	51
							Left middle frontal gyrus, orbital part, BA 46	17
							Left inferior frontal gyrus, orbital part, BA 47	16
							Left inferior frontal gyrus, orbital part	13
							Left anterior thalamic projections	9
							Left middle frontal gyrus, orbital part	5
							Left striatum	2

B, bilateral; L, left; MNI, Montreal Neurological Institute; R, right; SDM, Seed-based d Mapping.

### 3.4 DTI meta-analysis

The SDM-PSI-based meta-analysis of diffusion tensor imaging (DTI) data preliminarily suggested limited white matter microstructural alterations in post-stroke cognitive impairment (PSCI) patients at uncorrected thresholds (*p* < 0.05), as illustrated in Figure 2B and Table 3. In uncorrected analyses, the Left anterior cingulate/paracingulate gyri (BA 11, *p* = 0.033) demonstrated a trend toward reduced white matter integrity. Additionally, concurrent microstructural abnormalities were observed in the right median cingulate (*p* = 0.019) and left pons (*p* = 0.047). However, none of these white matter alterations reached statistical significance after rigorous multiple comparisons correction.

**TABLE 3:** Meta-analyses results regarding DTI difference between PSCI and control group.

Local maximum	Peak MNI coordinate (x, y, z)	SDM-Z value	*p*-value	No. of voxels	Egger’s test (*p*-value)	*I* ^2^	Cluster breakdown	
							**Brain region**	**No. of voxels**
***p* < 0.05 uncorrected**								
Right median cingulate	2, −20, 28	−2.074	0.019	244	*P* < 0.001	0.28 (*I*^2^< 50%)	Corpus callosum	57
							Right median cingulate/paracingulate gyri, BA 23	44
							Left median cingulate/paracingulate gyri, BA 23	27
							Left median network, cingulum	24
							Right median network, cingulum	17
							Left median cingulate/paracingulate gyri	10
							Right median cingulate/paracingulate gyri	9
Left anterior cingulate/paracingulate gyri, BA 11/25	−6, 26, −8	−1.836	0.033	46	*P* < 0.001	4.50 (I2 < 50%)	Left anterior cingulate/paracingulate gyri, BA 11/25	15
							Left olfactory cortex, BA 11	9
							Corpus callosum	9
							Left superior frontal gyrus, medial orbital, BA 11	6
							Left anterior cingulate/paracingulate gyri, BA 25	3
							Left olfactory cortex, BA 25	3
Left pons	−22, −16, −10	−1.669	0.047	12	*P* < 0.001	9.99 (*I*^2^< 50%)	Left pons	4
							Left hippocampus	3
							Left optic radiations	1

B, bilateral; L, left; MNI, Montreal Neurological Institute; R, right; SDM, Seed-based d Mapping.

### 3.5 Multimodal analysis

The integrated analysis of gray matter volume (GMV) atrophy and white matter diffusion tensor imaging (DTI) abnormalities, as illustrated in Figure 2C and Table 4, identified the left anterior cingulate/paracingulate gyri (BA 11/25) as exhibiting concurrent GMV reduction and white matter microstructural impairment. As a pivotal hub within the limbic system, this GM-WM covarying pathology may indicate a potential localized GM-WM covarying injury pattern, consequently contributing to emotional processing and cognitive control dysfunction in PSCI patients.

**TABLE 4:** Overlapping of GMV and DTI differences between PSCI and control group.

Local maximum	Cluster					
Region	Peak MNI coordinate (x, y, z)	SDM-Z value	*p*-value	No. of voxels	Local peaks and cluster breakdown	(No. of voxels)
***p* < 0.05 uncorrected**						
**Overlapping of GMV and FA**						
Left anterior cingulate/paracingulate gyri, BA 11/25	−6, 28, −10	−1.707	0.043	18	Left anterior cingulate/paracingulate gyri, BA 11/25	11
					Left superior frontal gyrus, medial orbital, BA 11	5
					Left olfactory cortex, BA 11	2

B, bilateral; L, left; MNI, Montreal Neurological Institute; R, right; SDM, Seed-based d Mapping.

#### 3.6 Analyses of heterogeneity and publication bias

In the final meta-analysis of studies investigating post-stroke cognitive impairment (PSCI), although heterogeneity was low (*I*^2^ < 50%), Egger’s test indicated significant publication bias for atrophy in the right temporal pole (*p* < 0.001) and white matter alterations (*p* < 0.001), suggesting potential overrepresentation of positive findings (Tables 2, 3).

#### 3.7 Meta-regression analyses

**FIGURE 3:**
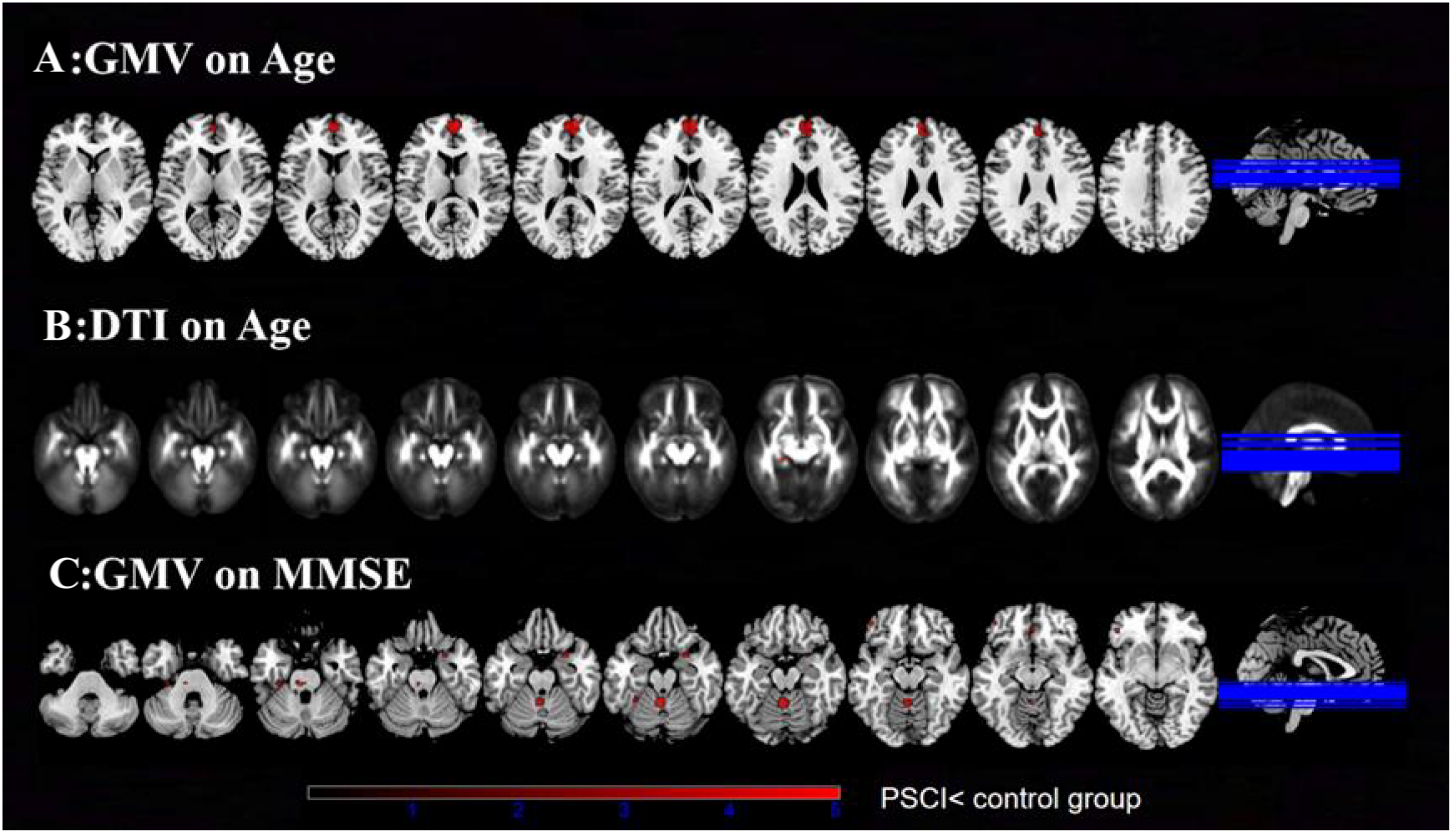
Meta-regression analyses. **(A)** Meta-regression analysis of GMV in PSCI on age. **(B)** Meta-regression analysis of DTI-FA (FA) in PSCI on age. **(C)** Meta-regression analysis of GMV in PSCI on MMSE. PSCI, post-stroke cognitive impairment; GMV, gray matter volume; DTI, diffusion tensor imaging; FA, fractional anisotropy; MMSE, Mini-Mental State Examination.

The current meta-regression analysis revealed significant negative correlations between age and GMV in the right medial superior frontal gyrus (BA 10, *p* = 0.006), and age and white matter connectivity in the left median cingulate network (*p* = 0.02, corrected). After rigorous correction, MMSE scores showed no significant associations with PSCI-related alterations. However, at uncorrected thresholds (*p* < 0.05), MMSE scores demonstrated negative correlations with structural measures in 17 brain regions including: cerebellar vermis lobules IV/V, left arcuate fasciculus (posterior segment), and right caudate nucleus, but showed no significant associations with white matter microstructure (Figure 3 and Supplementary Tables 8–10).

## 4 Discussion

This study applied voxel-based SDM-PSI multimodal meta-analysis to systematically characterize gray matter-white matter (GM-WM) covarying pathology in post-stroke cognitive impairment (PSCI). Key findings include significant GM volume atrophy in the right medial superior frontal gyrus (BA 10, showing the most extensive atrophy), right temporal pole (BA 38), and left orbitofrontal cortex (BA 47) and preliminary evidence of concurrent GM volume reduction and WM FA decrease in the left anterior cingulate/paracingulate gyri (BA 11/25). Meta-regression analyses further identified age as a significant modulator of right medial superior frontal gyrus (BA 10) atrophy, while the subclinical-stage BA 11 abnormalities highlight its relevance as a candidate region for investigating early neurodegenerative progression. These multimodal findings provide hypothesis-generating insights into PSCI’s “stroke lesion-remote area cascade” pathology, advancing both our understanding of neural network degeneration and the development of targeted neuromodulation interventions with anatomical guidance.

This multimodal neuroimaging meta-analysis systematically elucidates characteristic neuroanatomical alterations and their mechanistic associations with cognitive impairment in post-stroke cognitive impairment (PSCI). Neuroimaging analyses revealed functionally specific gray matter atrophy patterns, including significant volume reduction in the right medial superior frontal gyrus (BA 10; FWE-corrected *p* < 0.001), which showed strong associations with prefrontal executive network dysfunction, Left orbitofrontal cortex (BA 47; FWE-corrected *p* = 0.018) atrophy aligned with Pantoni’s findings on frontal-subcortical degeneration and executive deficits (Pantoni, 2010), further corroborated by Sundar et al.’s KCB (Sundar et al., 2023) statistical analysis of late-stage PSCI. Another study has found that subcortical ischemia in the frontal lobe is a key factor in post-stroke cognitive impairment (Kandiah et al., 2011). Degenerative changes in the right temporal pole (BA 38; FWE-corrected *p* = 0.003) primarily impacted socio-cognitive and semantic memory processing, consistent with Huai et al.’s observations (Huai et al., 2024) of reduced neural activity in the right superior temporal gyrus and precentral gyrus in PSCI patients.

Of particular interest, our study identifies the left anterior cingulate/paracingulate region (BA 11/25) as a potentially relevant neural hub in PSCI pathogenesis. Notably, this region showed significant GMV reduction (FWE-corrected), and an exploratory GM–WM overlap with reduced white matter microstructural integrity was observed at uncorrected thresholds. It is important to emphasize that while this overlapping cluster was focal (18 voxels) and did not survive stringent FWE correction, we interpret this spatial convergence as exploratory evidence suggesting a potential localized GM-WM covarying injury pattern. Consistent with this preliminary observation, Cai et al. (2023) reported compensatory neural hyperactivity in the right cingulate gyrus of PSCI patients, which aligns with our results. The anterior cingulate cortex (ACC) plays a pivotal role in cognitive and emotional processing (Bush et al., 2000; Journée et al., 2023; To et al., 2017) and has been implicated in schizophrenia (Huang et al., 2024). Specifically, studies have demonstrated that reduced functional connectivity between the ACC and dorsolateral prefrontal cortex (dlPFC) in schizophrenia patients is associated with executive dysfunction (Fortier et al., 2023). Furthermore, research has shown that cognitive impairment induced by occupational aluminum exposure correlates with the decoupling of gray matter and white matter functional networks (Zhang et al., 2024). In patients with white matter lesions (WMLs), investigations have revealed significant associations between structural volume alterations and cognitive assessment scores (Wang et al., 2019). Viewed through this lens, our findings tentatively suggest that the co-occurring glutamatergic neuronal degeneration in the dorsal ACC and axonal pathology in the ventral cingulum bundle may reflect a dual-pathology mechanism. This disruption could potentially impair neural signaling efficiency in cognitive control circuits and disrupts functional connectivity in emotional regulation networks (Bush et al., 2000).

Our meta-regression analysis revealed distinct patterns of structural neurodegeneration in post-stroke cognitive impairment (PSCI) modulated by key clinical variables. Most notably, we identified a significant negative association between advancing age and GMV in the right medial superior frontal gyrus (BA 10, *p* = 0.006), which aligns with previous research indicating a significant negative correlation between advancing age and gray matter atrophy in this region (Pan et al., 2023). This finding substantiates the brain reserve hypothesis, suggesting that older age exacerbates the severity of post-stroke neurodegenerative progression. Interestingly, while Mini-Mental State Examination (MMSE) scores showed expected strong correlations with global cognitive status, they failed to demonstrate significant associations with region-specific structural changes after false discovery rate correction (all *p* > 0.05). Rather than indicating a specific pathophysiological feature, this absence of significant correlation likely reflects the MMSE’s well-documented limitations, particularly its “ceiling effect” and lack of sensitivity to mild cognitive impairment and executive dysfunction—domains critically subserved by the prefrontal-limbic circuits identified in our study (Weaver et al., 2021). Consequently, the MMSE may fail to capture subtle cognitive deficits associated with the observed focal atrophy. Future studies should prioritize more domain-sensitive neuropsychological tools, such as the Montreal Cognitive Assessment (MoCA) for general screening or the Trail Making Test for executive function, to better elucidate structure-function relationships in PSCI (Pendlebury et al., 2010). These findings highlight the importance of selecting appropriate cognitive measures when studying neuroanatomical correlates of PSCI (Salvadori et al., 2021).

Several methodological limitations warrant discussion in this study. A primary constraint is the relatively small number of eligible studies (7 GMV and 8 DTI studies). While this sample size reflects the strict inclusion criteria and the current state of high-quality neuroimaging research in PSCI, it inherently limits the statistical power and generalizability of our findings. Consequently, the reported gray matter and white matter alterations should be interpreted as preliminary characterizations requiring validation in larger, multi-center cohorts. Beyond sample size, the reliance on coordinate-based meta-analytic approaches—integrating peak coordinates rather than raw imaging data—may marginally compromise spatial precision. This is particularly relevant for the observed GM-WM overlap in the ACC, which represents a convergence of statistical peaks rather than a verified voxel-to-voxel correspondence. Third, the white matter abnormalities and multimodal covariance findings were based on uncorrected thresholds. While this approach minimizes Type II errors in small-sample meta-analyses, these results should be interpreted with caution and require replication in larger cohorts with stringent multiple comparison corrections. We also observed significant Egger’s test results for the right temporal pole and DTI findings, highlighting a potential risk of publication bias where null results might be underrepresented. This could inflate effect sizes in these regions. While we rigorously adhered to PRISMA guidelines and employed SDM-PSI—an advanced meta-analytic method—to enhance result reliability, technical heterogeneity may persist due to variations in imaging acquisition parameters (e.g., MRI field strength, voxel size) and processing pipelines (e.g., smoothing kernels, normalization methods) across included studies. Notably, heterogeneity testing (*I*^2^< 50%) and sensitivity analyses confirmed the robustness of primary findings. Furthermore, the exclusive inclusion of peer-reviewed publications publications employing whole-brain analyses may introduce selection bias by excluding non-English literature, unpublished datasets, and studies utilizing alternative analytical strategies. Future investigations should prioritize comprehensive analyses of neuroimaging data from globally diverse populations to achieve more inclusive understanding. Additionally, we encountered technical constraints due to deprecated software modules in updated versions, limiting our capacity to conduct certain sensitivity analyses. Despite outreach to the software developers, no resolution has been obtained to date. We remain committed to addressing this limitation to enable more exhaustive data analyses in subsequent research.

## 5 Conclusion

This multimodal neuroimaging meta-analysis systematically characterizes distinctive neuropathological alterations in post-stroke cognitive impairment (PSCI). Key findings demonstrate: (1) significant gray matter atrophy in critical cognitive regions including the right medial superior frontal gyrus (BA 10), right temporal pole (BA 38), and left orbitofrontal cortex (BA 47); (2) exploratory evidence pointing to potential covarying gray matter volume reduction with white matter microstructural integrity loss in the left anterior cingulate/paracingulate region (BA 11/25); (3) Meta-regression analyses further revealed a significant negative association between advancing age and right medial superior frontal gyrus atrophy (*p* = 0.006), whereas MMSE scores showed no statistically significant correlations with region-specific structural changes. These findings may help guide future research aiming to identify objective biomarkers for early identification of high-risk patients and development of personalized intervention strategies in clinical practice.

## Data Availability

The datasets presented in this study can be found in online repositories. The names of the repository/repositories and accession number(s) can be found in this article/Supplementary material.

## Footnotes

1
https://www.jove.com/video/59841/

2
https://www.nitrc.org/frs/?group_id=152
