# Supplementary material for: Gray-white matter covarying impairment in the anterior cingulate cortex revealed by multimodal meta-analysis of post-stroke cognitive impairment
Source: Front Aging Neurosci. 2026 Feb 23;18:1654758. doi: 10.3389/fnagi.2026.1654758 (PMC12968202; doi:10.3389/fnagi.2026.1654758)
Supplement: Supplementary file 1 [file Data_Sheet_1.docx]

**Supplementary Table 1** The complete search formulas for PubMed

| **Pubmed** | | Total |
| --- | --- | --- |
| #1 | "Stroke"[MeSH Terms] OR ("Stroke"[MeSH Terms] OR "Stroke"[All Fields] OR ("cerebrovascular"[All Fields] AND "accident"[All Fields]) OR "cerebrovascular accident"[All Fields] OR ("Stroke"[MeSH Terms] OR "Stroke"[All Fields] OR ("cerebrovascular"[All Fields] AND "accidents"[All Fields]) OR "cerebrovascular accidents"[All Fields]) OR ("Stroke"[MeSH Terms] OR "Stroke"[All Fields] OR ("cerebral"[All Fields] AND "Stroke"[All Fields]) OR "cerebral stroke"[All Fields]) OR ("Stroke"[MeSH Terms] OR "Stroke"[All Fields] OR ("cerebral"[All Fields] AND "strokes"[All Fields]) OR "cerebral strokes"[All Fields]) OR ("Stroke"[MeSH Terms] OR "Stroke"[All Fields] OR ("Stroke"[All Fields] AND "cerebral"[All Fields]) OR "stroke cerebral"[All Fields]) OR ("Stroke"[MeSH Terms] OR "Stroke"[All Fields] OR ("strokes"[All Fields] AND "cerebral"[All Fields]) OR "strokes cerebral"[All Fields]) OR ("Stroke"[MeSH Terms] OR "Stroke"[All Fields] OR ("cerebrovascular"[All Fields] AND "apoplexy"[All Fields]) OR "cerebrovascular apoplexy"[All Fields]) OR ("Stroke"[MeSH Terms] OR "Stroke"[All Fields] OR ("apoplexy"[All Fields] AND "cerebrovascular"[All Fields]) OR "apoplexy cerebrovascular"[All Fields]) OR ("Stroke"[MeSH Terms] OR "Stroke"[All Fields] OR ("vascular"[All Fields] AND "accident"[All Fields] AND "brain"[All Fields]) OR "vascular accident brain"[All Fields]) OR ("Stroke"[MeSH Terms] OR "Stroke"[All Fields] OR ("brain"[All Fields] AND "vascular"[All Fields] AND "accident"[All Fields]) OR "brain vascular accident"[All Fields]) OR ("Stroke"[MeSH Terms] OR "Stroke"[All Fields] OR ("brain"[All Fields] AND "vascular"[All Fields] AND "accidents"[All Fields]) OR "brain vascular accidents"[All Fields]) OR ("Stroke"[MeSH Terms] OR "Stroke"[All Fields] OR ("vascular"[All Fields] AND "accidents"[All Fields] AND "brain"[All Fields])) OR ("Stroke"[MeSH Terms] OR "Stroke"[All Fields] OR ("cerebrovascular"[All Fields] AND "Stroke"[All Fields]) OR "cerebrovascular stroke"[All Fields]) OR ("Stroke"[MeSH Terms] OR "Stroke"[All Fields] OR ("cerebrovascular"[All Fields] AND "strokes"[All Fields]) OR "cerebrovascular strokes"[All Fields]) OR ("Stroke"[MeSH Terms] OR "Stroke"[All Fields] OR ("Stroke"[All Fields] AND "cerebrovascular"[All Fields]) OR "stroke cerebrovascular"[All Fields]) OR ("Stroke"[MeSH Terms] OR "Stroke"[All Fields] OR ("strokes"[All Fields] AND "cerebrovascular"[All Fields]) OR "strokes cerebrovascular"[All Fields]) OR ("apoplexies"[All Fields] OR "Stroke"[MeSH Terms] OR "Stroke"[All Fields] OR "apoplexy"[All Fields]) OR (("Stroke"[MeSH Terms] OR "Stroke"[All Fields] OR "cva"[All Fields]) AND ("Stroke"[MeSH Terms] OR "Stroke"[All Fields] OR ("cerebrovascular"[All Fields] AND "accident"[All Fields]) OR "cerebrovascular accident"[All Fields])) OR ("CVAs"[All Fields] AND ("Stroke"[MeSH Terms] OR "Stroke"[All Fields] OR ("cerebrovascular"[All Fields] AND "accident"[All Fields]) OR "cerebrovascular accident"[All Fields])) OR ("Stroke"[MeSH Terms] OR "Stroke"[All Fields] OR ("Stroke"[All Fields] AND "acute"[All Fields]) OR "stroke acute"[All Fields]) OR ("Stroke"[MeSH Terms] OR "Stroke"[All Fields] OR ("acute"[All Fields] AND "Stroke"[All Fields]) OR "acute stroke"[All Fields]) OR ("Stroke"[MeSH Terms] OR "Stroke"[All Fields] OR ("acute"[All Fields] AND "strokes"[All Fields]) OR "acute strokes"[All Fields]) OR ("Stroke"[MeSH Terms] OR "Stroke"[All Fields] OR ("strokes"[All Fields] AND "acute"[All Fields]) OR "strokes acute"[All Fields]) OR ("Stroke"[MeSH Terms] OR "Stroke"[All Fields] OR ("cerebrovascular"[All Fields] AND "accident"[All Fields] AND "acute"[All Fields]) OR "cerebrovascular accident acute"[All Fields]) OR ("Stroke"[MeSH Terms] OR "Stroke"[All Fields] OR ("acute"[All Fields] AND "cerebrovascular"[All Fields] AND "accident"[All Fields]) OR "acute cerebrovascular accident"[All Fields]) OR ("Stroke"[MeSH Terms] OR "Stroke"[All Fields] OR ("acute"[All Fields] AND "cerebrovascular"[All Fields] AND "accidents"[All Fields]) OR "acute cerebrovascular accidents"[All Fields]) OR ("Stroke"[MeSH Terms] OR "Stroke"[All Fields] OR ("cerebrovascular"[All Fields] AND "accidents"[All Fields] AND "acute"[All Fields]) OR "cerebrovascular accidents acute"[All Fields])) |  |
| #2 | "Cognitive Dysfunction"[MeSH Terms] OR ("Cognitive Dysfunction"[MeSH Terms] OR ("cognitive"[All Fields] AND "dysfunction"[All Fields]) OR "Cognitive Dysfunction"[All Fields] OR ("Cognitive Dysfunction"[MeSH Terms] OR ("cognitive"[All Fields] AND "dysfunction"[All Fields]) OR "Cognitive Dysfunction"[All Fields] OR ("dysfunction"[All Fields] AND "cognitive"[All Fields]) OR "dysfunction cognitive"[All Fields]) OR ("Cognitive Dysfunction"[MeSH Terms] OR ("cognitive"[All Fields] AND "dysfunction"[All Fields]) OR "Cognitive Dysfunction"[All Fields] OR ("dysfunctions"[All Fields] AND "cognitive"[All Fields]) OR "dysfunctions cognitive"[All Fields]) OR ("Cognitive Dysfunction"[MeSH Terms] OR ("cognitive"[All Fields] AND "dysfunction"[All Fields]) OR "Cognitive Dysfunction"[All Fields] OR ("cognitive"[All Fields] AND "disorder"[All Fields]) OR "cognitive disorder"[All Fields]) OR ("Cognitive Dysfunction"[MeSH Terms] OR ("cognitive"[All Fields] AND "dysfunction"[All Fields]) OR "Cognitive Dysfunction"[All Fields] OR ("cognitive"[All Fields] AND "disorders"[All Fields]) OR "cognitive disorders"[All Fields]) OR ("Cognitive Dysfunction"[MeSH Terms] OR ("cognitive"[All Fields] AND "dysfunction"[All Fields]) OR "Cognitive Dysfunction"[All Fields] OR ("disorder"[All Fields] AND "cognitive"[All Fields]) OR "disorder cognitive"[All Fields]) OR ("Cognitive Dysfunction"[MeSH Terms] OR ("cognitive"[All Fields] AND "dysfunction"[All Fields]) OR "Cognitive Dysfunction"[All Fields] OR ("disorders"[All Fields] AND "cognitive"[All Fields]) OR "disorders cognitive"[All Fields]) OR ("Cognitive Dysfunction"[MeSH Terms] OR ("cognitive"[All Fields] AND "dysfunction"[All Fields]) OR "Cognitive Dysfunction"[All Fields] OR ("cognitive"[All Fields] AND "impairments"[All Fields]) OR "cognitive impairments"[All Fields]) OR ("Cognitive Dysfunction"[MeSH Terms] OR ("cognitive"[All Fields] AND "dysfunction"[All Fields]) OR "Cognitive Dysfunction"[All Fields] OR ("cognitive"[All Fields] AND "impairment"[All Fields]) OR "cognitive impairment"[All Fields]) OR ("Cognitive Dysfunction"[MeSH Terms] OR ("cognitive"[All Fields] AND "dysfunction"[All Fields]) OR "Cognitive Dysfunction"[All Fields] OR ("impairment"[All Fields] AND "cognitive"[All Fields]) OR "impairment cognitive"[All Fields]) OR ("Cognitive Dysfunction"[MeSH Terms] OR ("cognitive"[All Fields] AND "dysfunction"[All Fields]) OR "Cognitive Dysfunction"[All Fields] OR ("impairments"[All Fields] AND "cognitive"[All Fields]) OR "impairments cognitive"[All Fields]) OR ("Cognitive Dysfunction"[MeSH Terms] OR ("cognitive"[All Fields] AND "dysfunction"[All Fields]) OR "Cognitive Dysfunction"[All Fields] OR ("mild"[All Fields] AND "cognitive"[All Fields] AND "impairment"[All Fields]) OR "mild cognitive impairment"[All Fields]) OR ("Cognitive Dysfunction"[MeSH Terms] OR ("cognitive"[All Fields] AND "dysfunction"[All Fields]) OR "Cognitive Dysfunction"[All Fields] OR ("cognitive"[All Fields] AND "impairment"[All Fields] AND "mild"[All Fields]) OR "cognitive impairment mild"[All Fields]) OR ("Cognitive Dysfunction"[MeSH Terms] OR ("cognitive"[All Fields] AND "dysfunction"[All Fields]) OR "Cognitive Dysfunction"[All Fields] OR ("cognitive"[All Fields] AND "impairments"[All Fields] AND "mild"[All Fields]) OR "cognitive impairments mild"[All Fields]) OR ("Cognitive Dysfunction"[MeSH Terms] OR ("cognitive"[All Fields] AND "dysfunction"[All Fields]) OR "Cognitive Dysfunction"[All Fields] OR ("impairment"[All Fields] AND "mild"[All Fields] AND "cognitive"[All Fields]) OR "impairment mild cognitive"[All Fields]) OR ("Cognitive Dysfunction"[MeSH Terms] OR ("cognitive"[All Fields] AND "dysfunction"[All Fields]) OR "Cognitive Dysfunction"[All Fields] OR ("impairments"[All Fields] AND "mild"[All Fields] AND "cognitive"[All Fields]) OR "impairments mild cognitive"[All Fields]) OR ("Cognitive Dysfunction"[MeSH Terms] OR ("cognitive"[All Fields] AND "dysfunction"[All Fields]) OR "Cognitive Dysfunction"[All Fields] OR ("mild"[All Fields] AND "cognitive"[All Fields] AND "impairments"[All Fields]) OR "mild cognitive impairments"[All Fields]) OR ("Cognitive Dysfunction"[MeSH Terms] OR ("cognitive"[All Fields] AND "dysfunction"[All Fields]) OR "Cognitive Dysfunction"[All Fields] OR ("cognitive"[All Fields] AND "decline"[All Fields]) OR "cognitive decline"[All Fields]) OR ("Cognitive Dysfunction"[MeSH Terms] OR ("cognitive"[All Fields] AND "dysfunction"[All Fields]) OR "Cognitive Dysfunction"[All Fields] OR ("cognitive"[All Fields] AND "declines"[All Fields]) OR "cognitive declines"[All Fields]) OR ("Cognitive Dysfunction"[MeSH Terms] OR ("cognitive"[All Fields] AND "dysfunction"[All Fields]) OR "Cognitive Dysfunction"[All Fields] OR ("decline"[All Fields] AND "cognitive"[All Fields]) OR "decline cognitive"[All Fields]) OR ("Cognitive Dysfunction"[MeSH Terms] OR ("cognitive"[All Fields] AND "dysfunction"[All Fields]) OR "Cognitive Dysfunction"[All Fields] OR ("declines"[All Fields] AND "cognitive"[All Fields]) OR "declines cognitive"[All Fields]) OR ("Cognitive Dysfunction"[MeSH Terms] OR ("cognitive"[All Fields] AND "dysfunction"[All Fields]) OR "Cognitive Dysfunction"[All Fields] OR ("mental"[All Fields] AND "deterioration"[All Fields]) OR "mental deterioration"[All Fields]) OR ("Cognitive Dysfunction"[MeSH Terms] OR ("cognitive"[All Fields] AND "dysfunction"[All Fields]) OR "Cognitive Dysfunction"[All Fields] OR ("deterioration"[All Fields] AND "mental"[All Fields]) OR "deterioration mental"[All Fields]) OR ("Cognitive Dysfunction"[MeSH Terms] OR ("cognitive"[All Fields] AND "dysfunction"[All Fields]) OR "Cognitive Dysfunction"[All Fields] OR ("deteriorations"[All Fields] AND "mental"[All Fields])) OR ("Cognitive Dysfunction"[MeSH Terms] OR ("cognitive"[All Fields] AND "dysfunction"[All Fields]) OR "Cognitive Dysfunction"[All Fields] OR ("mental"[All Fields] AND "deteriorations"[All Fields]) OR "mental deteriorations"[All Fields])) |  |
| #3 | ((("Gray Matter"[Mesh]) OR (((((((((((((((((((Gray Matters) OR (Matter, Gray)) OR (Matters, Gray)) OR (Grey Matter)) OR (Grey Matters)) OR (Matter, Grey)) OR (Matters, Grey)) OR (Cerebellar Gray Matter)) OR (Cerebellar Gray Matters)) OR (Gray Matter, Cerebellar)) OR (Gray Matters, Cerebellar)) OR (Matter, Cerebellar Gray)) OR (Matters, Cerebellar Gray)) OR (Cerebellar Grey Matter)) OR (Cerebellar Grey Matters)) OR (Grey Matter, Cerebellar)) OR (Grey Matters, Cerebellar)) OR (Matter, Cerebellar Grey)) OR (Matters, Cerebellar Grey))) AND (("Cognitive Dysfunction"[Mesh]) OR (((((((((((((((((((((((((Cognitive Dysfunction) OR (Dysfunction, Cognitive)) OR (Dysfunctions, Cognitive)) OR (Cognitive Disorder)) OR (Cognitive Disorders)) OR (Disorder, Cognitive)) OR (Disorders, Cognitive)) OR (Cognitive Impairments)) OR (Cognitive Impairment)) OR (Impairment, Cognitive)) OR (Impairments, Cognitive)) OR (Mild Cognitive Impairment)) OR (Cognitive Impairment, Mild)) OR (Cognitive Impairments, Mild)) OR (Impairment, Mild Cognitive)) OR (Impairments, Mild Cognitive)) OR (Mild Cognitive Impairments)) OR (Cognitive Decline)) OR (Cognitive Declines)) OR (Decline, Cognitive)) OR (Declines, Cognitive)) OR (Mental Deterioration)) OR (Deterioration, Mental)) OR (Deteriorations, Mental)) OR (Mental Deteriorations)))) AND (((((((((((((((((((((((((((Cerebrovascular Accident) OR (Cerebrovascular Accidents)) OR (Cerebral Stroke)) OR (Cerebral Strokes)) OR (Stroke, Cerebral)) OR (Strokes, Cerebral)) OR (Cerebrovascular Apoplexy)) OR (Apoplexy, Cerebrovascular)) OR (Vascular Accident, Brain)) OR (Brain Vascular Accident)) OR (Brain Vascular Accidents)) OR (Vascular Accidents, Brain)) OR (Cerebrovascular Stroke)) OR (Cerebrovascular Strokes)) OR (Stroke, Cerebrovascular)) OR (Strokes, Cerebrovascular)) OR (Apoplexy)) OR (CVA (Cerebrovascular Accident))) OR (CVAs (Cerebrovascular Accident))) OR (Stroke, Acute)) OR (Acute Stroke)) OR (Acute Strokes)) OR (Strokes, Acute)) OR (Cerebrovascular Accident, Acute)) OR (Acute Cerebrovascular Accident)) OR (Acute Cerebrovascular Accidents)) OR (Cerebrovascular Accidents, Acute)) |  |
| #4 | "Diffusion Tensor Imaging"[MeSH Terms] OR ("Diffusion Tensor Imaging"[MeSH Terms] OR ("diffusion"[All Fields] AND "tensor"[All Fields] AND "imaging"[All Fields]) OR "Diffusion Tensor Imaging"[All Fields] OR ("imaging"[All Fields] AND "diffusion"[All Fields] AND "tensor"[All Fields]) OR "imaging diffusion tensor"[All Fields] OR ("Diffusion Tensor Imaging"[MeSH Terms] OR ("diffusion"[All Fields] AND "tensor"[All Fields] AND "imaging"[All Fields]) OR "Diffusion Tensor Imaging"[All Fields] OR ("diffusion"[All Fields] AND "tensor"[All Fields] AND "mri"[All Fields]) OR "diffusion tensor mri"[All Fields]) OR ("Diffusion Tensor Imaging"[MeSH Terms] OR ("diffusion"[All Fields] AND "tensor"[All Fields] AND "imaging"[All Fields]) OR "Diffusion Tensor Imaging"[All Fields]) OR ("Diffusion Tensor Imaging"[MeSH Terms] OR ("diffusion"[All Fields] AND "tensor"[All Fields] AND "imaging"[All Fields]) OR "Diffusion Tensor Imaging"[All Fields] OR ("diffusion"[All Fields] AND "tensor"[All Fields] AND "mris"[All Fields]) OR "diffusion tensor mris"[All Fields]) OR ("Diffusion Tensor Imaging"[MeSH Terms] OR ("diffusion"[All Fields] AND "tensor"[All Fields] AND "imaging"[All Fields]) OR "Diffusion Tensor Imaging"[All Fields] OR ("mri"[All Fields] AND "diffusion"[All Fields] AND "tensor"[All Fields]) OR "mri diffusion tensor"[All Fields]) OR ("Diffusion Tensor Imaging"[MeSH Terms] OR ("diffusion"[All Fields] AND "tensor"[All Fields] AND "imaging"[All Fields]) OR "Diffusion Tensor Imaging"[All Fields] OR ("dti"[All Fields] AND "mri"[All Fields]) OR "dti mri"[All Fields]) OR ("Diffusion Tensor Imaging"[MeSH Terms] OR ("diffusion"[All Fields] AND "tensor"[All Fields] AND "imaging"[All Fields]) OR "Diffusion Tensor Imaging"[All Fields] OR ("diffusion"[All Fields] AND "tensor"[All Fields] AND "magnetic"[All Fields] AND "resonance"[All Fields] AND "imaging"[All Fields]) OR "diffusion tensor magnetic resonance imaging"[All Fields]) OR ("Diffusion Tensor Imaging"[MeSH Terms] OR ("diffusion"[All Fields] AND "tensor"[All Fields] AND "imaging"[All Fields]) OR "Diffusion Tensor Imaging"[All Fields] OR ("diffusion"[All Fields] AND "tractography"[All Fields]) OR "diffusion tractography"[All Fields]) OR ("Diffusion Tensor Imaging"[MeSH Terms] OR ("diffusion"[All Fields] AND "tensor"[All Fields] AND "imaging"[All Fields]) OR "Diffusion Tensor Imaging"[All Fields] OR ("tractography"[All Fields] AND "diffusion"[All Fields]) OR "tractography diffusion"[All Fields])) |  |
| #5 | #1 AND #2 AND #3 | 460 |
| #6 | #1 AND #2 AND #4 | 374 |

**Supplementary Table 2** The complete search formulas for Embase

| **Embase** | | Total |
| --- | --- | --- |
| #1 | strokes OR (cerebrovascular AND accidents) OR (cerebral AND stroke) OR (cerebral AND strokes) OR (stroke, AND cerebral) OR (strokes, AND cerebral) OR (cerebrovascular AND apoplexy) OR (apoplexy, AND cerebrovascular) OR (vascular AND accident, AND brain) OR (brain AND vascular AND accident) OR (brain AND vascular AND accidents) OR (vascular AND accidents, AND brain) OR (cerebrovascular AND stroke) OR (cerebrovascular AND strokes) OR (stroke, AND cerebrovascular) OR (strokes, AND cerebrovascular) OR apoplexy OR (cva AND cerebrovascular AND accident) OR (cvas AND cerebrovascular AND accident) OR (stroke, AND acute) OR (acute AND strokes) OR (strokes, AND acute) OR (cerebrovascular AND accident, AND acute) OR (acute AND cerebrovascular AND accident) OR (acute AND cerebrovascular AND accidents) OR (cerebrovascular AND accidents, AND acute) |  |
| #2 | dysfunction, AND cognitive OR (cognitive AND dysfunction) OR (dysfunctions, AND cognitive) OR (cognitive AND disorder) OR (cognitive AND disorders) OR (disorder, AND cognitive) OR (disorders, AND cognitive) OR (cognitive AND impairments) OR (cognitive AND impairment) OR (impairment, AND cognitive) OR (impairments, AND cognitive) OR (mild AND cognitive AND impairment) OR (cognitive AND impairment, AND mild) OR (cognitive AND impairments, AND mild) OR (impairment, AND mild AND cognitive) OR (impairments, AND mild AND cognitive) OR (mild AND cognitive AND impairments) OR (cognitive AND decline) OR (cognitive AND declines) OR (decline, AND cognitive) OR (declines, AND cognitive) OR (mental AND deterioration) OR (deterioration, AND mental) OR (deteriorations, AND mental) OR (mental AND deteriorations) |  |
| #3 | gray AND matters OR (matter, AND gray) OR (matters, AND gray) OR (grey AND matter) OR (grey AND matters) OR (matter, AND grey) OR (matters, AND grey) OR (cerebellar AND gray AND matter) OR (cerebellar AND gray AND matters) OR (gray AND matter, AND cerebellar) OR (gray AND matters, AND cerebellar) OR (matter, AND cerebellar AND gray) OR (matters, AND cerebellar AND gray) OR (cerebellar AND grey AND matter) OR (cerebellar AND grey AND matters) OR (grey AND matter, AND cerebellar) OR (grey AND matters, AND cerebellar) OR (matter, AND cerebellar AND grey) OR (matters, AND cerebellar AND grey) |  |
| #4 | diffusion AND tensor AND imaging OR (imaging, AND diffusion AND tensor) OR (diffusion AND tensor AND mri) OR (diffusion AND tensor AND mris) OR (mri, AND diffusion AND tensor) OR (dti AND mri) OR (diffusion AND tensor AND magnetic AND resonance AND imaging) OR (diffusion AND tractography) OR (tractography, AND diffusion) |  |
| #5 | #1 AND #2 AND #3 | 731 |
| #6 | #1 AND #2 AND #4 | 637 |

**Supplementary Table 3** The complete search formulas for Web of Science

| **Web of Science** | | Total |
| --- | --- | --- |
| #1 | Strokes (Topic) or Cerebrovascular Accidents (Topic) or Cerebral Stroke (Topic) or Cerebral Strokes (Topic) or Stroke, Cerebral (Topic) or Strokes, Cerebral (Topic) or Cerebrovascular Apoplexy (Topic) or Apoplexy, Cerebrovascular (Topic) or Vascular Accident, Brain (Topic) or Brain Vascular Accident (Topic) or Brain Vascular Accidents (Topic) or Vascular Accidents, Brain (Topic) or Cerebrovascular Stroke (Topic) or Cerebrovascular Strokes (Topic) or Stroke, Cerebrovascular (Topic) or Strokes, Cerebrovascular (Topic) or Apoplexy (Topic) or CVA (Cerebrovascular Accident) (Topic) or cmas (Cerebrovascular Accident) (Topic) or Stroke, Acute (Topic) or Acute Stroke (Topic) or Acute Strokes (Topic) or Strokes, Acute (Topic) or Cerebrovascular Accident, Acute (Topic) or Acute Cerebrovascular Accident (Topic) or Acute Cerebrovascular Accidents (Topic) or Cerebrovascular Accidents, Acute (Topic) and Preprint Citation Index (Exclude – Database) |  |
| #2 | Cognitive Dysfunction (Topic) or Dysfunction, Cognitive (Topic) or Dysfunctions, Cognitive (Topic) or Cognitive Disorder (Topic) or Cognitive Disorders (Topic) or Disorder, Cognitive (Topic) or Disorders, Cognitive (Topic) or Cognitive Impairments (Topic) or Cognitive Impairment (Topic) or Impairment, Cognitive (Topic) or Impairments, Cognitive (Topic) or Mild Cognitive Impairment (Topic) or Cognitive Impairment, Mild (Topic) or Cognitive Impairments, Mild (Topic) or Impairment, Mild Cognitive (Topic) or Impairments, Mild Cognitive (Topic) or Mild Cognitive Impairments (Topic) or Cognitive Decline (Topic) or Cognitive Declines (Topic) or Decline, Cognitive (Topic) or Declines, Cognitive (Topic) or Mental Deterioration (Topic) or Deterioration, Mental (Topic) or Deteriorations, Mental (Topic) or Mental Deteriorations (Topic) |  |
| #3 | CGray Matters (Topic) or Matter, Gray (Topic) or Matters, Gray (Topic) or Grey Matter (Topic) or Grey Matters (Topic) or Matter, Grey (Topic) or Matters, Grey (Topic) or Cerebellar Gray Matter (Topic) or Cerebellar Gray Matters (Topic) or Gray Matter, Cerebellar (Topic) or Gray Matters, Cerebellar (Topic) or Matter, Cerebellar Gray (Topic) or Matters, Cerebellar Gray (Topic) or Cerebellar Grey Matter (Topic) or Cerebellar Grey Matters (Topic) or Grey Matter, Cerebellar (Topic) or Grey Matters, Cerebellar (Topic) or Matter, Cerebellar Grey (Topic) or Matters, Cerebellar Grey (Topic) and Preprint Citation Index (Exclude – Database) |  |
| #4 | Diffusion Tensor Imaging (Topic) or Imaging, Diffusion Tensor (Topic) or Diffusion Tensor MRI (Topic) or Diffusion Tensor MRIs (Topic) or MRI, Diffusion Tensor (Topic) or DTI MRI (Topic) or Diffusion Tensor Magnetic Resonance Imaging (Topic) or Diffusion Tractography (Topic) or Tractography, Diffusion (Topic) and Preprint Citation Index (Exclude – Database) |  |
| #5 | #1 AND #2 AND #3 | 964 |
| #6 | #1 AND #2 AND #4 | 621 |

**Supplementary Table 4** Quality assessment checklist (score 0/0.5/1 per item; total score out of 10) *

| Category 1: Participants |
| --- |
| 1. Patients were evaluated prospectively, specific diagnostic criteria were applied, and demographic data were reported. |
| 2. Healthy comparison participants were evaluated prospectively, psychiatric and medical illnesses were excluded. |
| 3. Important variables (e.g., age, sex, illness duration, onset, medication status, comorbidity, severity of illness) were checked either by stratification or statistically. |
| 4. Sample size per group > 10. |
| Category 2: Methods for image acquisition and analysis |
| 5. Whole brain analysis was automated with no a priori regional selection. |
| 6. Coordinates reported in a standard space. |
| 7. The imaging technique used was clearly described so that it could be reproduced. |
| 8. Measurements were clearly described so that they could be reproduced. |
| Category 3: Results and conclusions |
| 9. Statistical parameters for significant and important nonsignificant differences were provided. |
| 10. Conclusions were consistent with the results obtained and the limitations were discussed. |
| *When criteria were partially met, 0.5 points were awarded. |

**Supplementary Table 5** Demographic, clinical and imaging characteristics of the included studies of GMV

| **study** | **PSCI** | | | | | **control group** | | | | | **Imaging characteristics** | | | | **Quality score** |
| --- | --- | --- | --- | --- | --- | --- | --- | --- | --- | --- | --- | --- | --- | --- | --- |
|  | **Partic**  **ipants**  **(male)** | **Age**  **(Y±SD)** | **MMSE** | **Total brain volume(×10³mm³)** | **Partic**  **iPants**  **(male)** | | **Age**  **(Y±SD)** | **MMSE** | **Total brain volume**  **（cm^3^）** | **FWHM** | | **Voxel**  **size(mm^3^)** | **Threshold(tfceCorrected)** |  | |
| (Grau-Olivares et al. 2010) | 15(7) | 71.7± 12.0 | 27.6±1.9 | 565.58± 69.85 | 15(11) | | 65.1± 11.3 | 29.5± 0.8 | 617.00± 85.83 | NA | | 1×1×1 | p<0.05 | 7 | |
| (Burton et al. 2003) | 50(21) | 79.8± 3.8 | 26.4± 2.3 | NA | 15(5) | | 81.3± 4.8 | 27.7± 1.7 | NA | 10 | | 2×2×2 | p<0.05 | 8.5 | |
| (Stebbins et al. 2008) | 40(19) | 52.9±4.2 | 25.8± 3.6 | 641.2±76.2 | 51(27) | | 63.1± 8.2 | 28.7± 1.1 | 608.3±99.9 | 8 | | 1×1×1 | p<0.05 | 8.5 | |
| (Luo et al. 2015) | 62(34) | 36.05±10.30 | 25.3± 0.7 | 615.5± 49.5 | 62(34) | | 53.1± 4.0 | 28.2± 0.6 | 670.5± 38.7 | 10 | | NA | p<0.01 | 8 | |
| (Lei et al. 2016) | 43(22) | 43.5± 14.1 | 26.5± 2.9 | NA | 55(30) | | 42.7± 12.1 | 29.3± 0.9 | NA | 8 | | 1×1×1 | p<0.05 | 8 | |
| (Tan et al. 2023) | 63(24) | 61.0±NA | 22±NA | 503.9± 51.2 | 14(6) | | 53.5±NA | 28±NA | 564.2± 63.6 | NA | | 1×1×1 | p<0.05 | 8.5 | |
| (Wang et al. 2023) | 19(13) | 58.3±6.4 | 19.4± 4.4 | NA | 19(13) | | 56.8± 6.0 | 27.9± 1.5 | NA | 8 | | 1.5×1.5×1.5 | p<0.05 | 9 | |

**Abbreviations:** PSCI, Post-Stroke Cognitive Impairment; GMV, Gray Matter Volume; FWHM, full width at half maximum; NA, not available; MMSE, Mini-Mental State Examination

**Supplementary Table 6** Demographic, clinical and imaging characteristics of the included studies of DTI

| **study** | **PSCI** | | | **control group** | | | **Imaging characteristics** | | | | **Quality score** |
| --- | --- | --- | --- | --- | --- | --- | --- | --- | --- | --- | --- |
|  | **Partic**  **ipants**  **(male)** | **Age**  **(Y±SD)** | **MMSE** | **Partic**  **iPants**  **(male)** | **Age**  **(Y±SD)** | **MMSE** | **FA** | **FWHM(mm)** | **Voxel**  **Size(mm^3^)** | **Threshold** |  |
| (Fernández-Andújar et al. 2014) | 17(13) | 62.4± 9.8 | NA | 17(13) | 61.7± 5.4 | NA | 1 | NA | 2×2×2 | p<0.05 | 8 |
| (Wei et al. 2021) | 33(22) | 57.6± 5.7 | NA | 30(16) | 57.0± 7.8 | NA | 1 | NA | 3×3×3 | p<0.05 | 7.5 |
| (Guo et al. 2014) | 35(22) | 51±NA | 24.3± 3.5 | 35(19) | 45±NA | 26.3± 1.2 | 1 | 8 | 3×3×3 | p<0.05 | 8.5 |
| (Wu et al. 2023) | 60(42) | 56.2± 7.0 | 28.8±1.1 | 25(16) | 57.7± 7.3 | 21.8± 5.2 | 1 | NA | 3×3×3 | p<0.05 | 8 |
| (Zhou et al. 2011) | 18(12) | 66.2± 7.7 | NA | 18(12) | 67.1± 7.8 | NA | 1 | 6 | 3×3×3 | p<0.05 | 8 |
| (Chen et al. 2025) | 41(20) | 55.2± 6.5 | 21.8± 4.1 | 41(23) | 56.2± 5.3 | 28.8± 1.1 | 2 | 8 | 3×3×3 | p<0.05 | 7 |
| (Dacosta-Aguayo et al. 2014) | 18(11) | 63±NA | 29.1± 1.3 | 8(6) | 66.5± 6.4 | 27.3± 2.5 | 1 | NA | 2×2×2 | p<0.02 | 7 |
| (Schaapsmeerders et al. 2016) | 117(47) | 49.8± 9.4 | NA | 84(38) | 48.9± 11.9 | NA | 1 | NA | 2×2×2 | p<0.05 | 8.5 |

**Abbreviations:** PSCI, Post-Stroke Cognitive Impairment; DTI, Diffusion Tensor Imaging;FA, Fractional Anisotropy; FWHM, full width at half maximum; NA, not available; MMSE, Mini-Mental State Examination

References

Burton, E., C. Ballard, S. Stephens, R. A. Kenny, R. Kalaria, R. Barber, and J. O'Brien. 2003. Hyperintensities and fronto-subcortical atrophy on MRI are substrates of mild cognitive deficits after stroke. DEMENTIA AND GERIATRIC COGNITIVE DISORDERS 16 (2):113-118.

Chen, M., Y. Wu, Y. Wang, and Z. Li. 2025. Functional connectivity and white matter microstructural alterations in patients with left basal ganglia acute ischemic stroke. Brain Imaging and Behavior 19 (2):421-432.

Dacosta-Aguayo, R., M. Graña, M. Fernández-Andújar, E. López-Cancio, C. Cáceres, N. Bargalló, M. Barrios, I. Clemente, P. T. Monserrat, M. A. Sas, A. Dávalos, T. Auer, and M. Mataró. 2014. Structural integrity of the contralesional hemisphere predicts cognitive impairment in ischemic stroke at three months. PLoS One 9 (1):e86119.

Fernández-Andújar, M., F. Doornink, R. Dacosta-Aguayo, J. J. Soriano-Raya, J. Miralbell, N. Bargalló, E. López-Cancio, D. L. O. N. Pérez, M. Gomis, M. Millán, M. Barrios, C. Cáceres, G. Pera, R. Forés, I. Clemente, A. Dávalos, and M. Mataró. 2014. Remote thalamic microstructural abnormalities related to cognitive function in ischemic stroke patients. NEUROPSYCHOLOGY 28 (6):984-996.

Grau-Olivares, M., A. Arboix, C. Junqué, E. M. Arenaza-Urquijo, M. Rovira, and D. Bartrés-Faz. 2010. Progressive gray matter atrophy in lacunar patients with vascular mild cognitive impairment. CEREBROVASCULAR DISEASES 30 (2):157-166.

Guo, J., S. Wang, R. Li, N. Chen, M. Zhou, H. Chen, Q. Gong, and L. He. 2014. Cognitive impairment and whole brain diffusion in patients with carotid artery disease and ipsilateral transient ischemic attack. NEUROLOGICAL RESEARCH 36 (1):41-46.

Lei, Y., J. Su, Q. Guo, H. Yang, Y. Gu, and Y. Mao. 2016. Regional Gray Matter Atrophy in Vascular Mild Cognitive Impairment. Journal of Stroke & Cerebrovascular Diseases 25 (1):95-101.

Luo, W., X. Jiang, X. Wei, S. Li, and M. Li. 2015. A study on cognitive impairment and gray matter volume abnormalities in silent cerebral infarction patients. NEURORADIOLOGY 57 (8):783-789.

Schaapsmeerders, P., A. M. Tuladhar, R. M. Arntz, S. Franssen, N. A. Maaijwee, L. C. Rutten-Jacobs, H. C. Schoonderwaldt, L. D. Dorresteijn, E. J. van Dijk, R. P. Kessels, and F. E. de Leeuw. 2016. Remote Lower White Matter Integrity Increases the Risk of Long-Term Cognitive Impairment After Ischemic Stroke in Young Adults. STROKE 47 (10):2517-2525.

Stebbins, G. T., D. L. Nyenhuis, C. Wang, J. L. Cox, S. Freels, K. Bangen, L. DeToledo-Morrell, K. Sripathirathan, M. Moseley, D. A. Turner, J. D. Gabrieli, and P. B. Gorelick. 2008. Gray matter atrophy in patients with ischemic stroke with cognitive impairment. STROKE 39 (3):785-793.

Tan, L., J. Xing, Z. Wang, Du X, R. Luo, J. Wang, J. Zhao, W. Zhao, and C. Yin. 2023. Study of gray matter atrophy pattern with subcortical ischemic vascular disease-vascular cognitive impairment no dementia based on structural magnetic resonance imaging. Frontiers in Aging Neuroscience 15:1051177.

Wang, H., M. Yu, J. Ren, X. Zhong, D. Xu, L. Gao, and H. Xu. 2023. Neuroanatomical correlates of cognitive impairment following basal ganglia-thalamic post-hemorrhagic stroke: Uncovering network-wide alterations in hemispheric gray matter asymmetry. BRAIN RESEARCH 1820:148559.

Wei, Y., C. Wang, J. Liu, P. Miao, S. Wei, Y. Wang, L. Wu, B. Xu, S. Han, Y. Wei, K. Wang, and J. Cheng. 2021. Widespread White Matter Microstructure Alterations Based on Diffusion Tensor Imaging and Diffusion Kurtosis Imaging in Patients With Pontine Infarction. Frontiers in Aging Neuroscience 13:758236.

Wu, B., S. Guo, X. Jia, Z. Geng, and Q. Yang. 2023. White Matter Microstructural Alterations over the Year after Acute Ischemic Stroke in Patients with Baseline Impaired Cognitive Functions. NEURAL PLASTICITY 2023:6762225.

Zhou, Y., Qun-Xu, L. D. Qin, L. J. Qian, W. W. Cao, and J. R. Xu. 2011. A primary study of diffusion tensor imaging-based histogram analysis in vascular cognitive impairment with no dementia. CLINICAL NEUROLOGY AND NEUROSURGERY 113 (2):92-97.

**Supplementary Table 7** Study-level effect sizes and pooled estimate for whole-brain GMV

| object of study |  | d |  | SE |  | Z |  | P |  | *CI*_low_ |  | *CI*_up_ |
| --- | --- | --- | --- | --- | --- | --- | --- | --- | --- | --- | --- | --- |
| (Grau-Olivares et al. 2010) |  | -0.641 |  | 0.376 |  | -1.706 |  | 0.0880 |  | -1.377 |  | 0.095 |
| (Stebbins et al. 2008) |  | -0.377 |  | 0.213 |  | -1.768 |  | 0.0770 |  | -0.795 |  | 0.041 |
| (Luo et al. 2015) |  | -1.247 |  | 0.197 |  | -6.336 |  | 0.0000 |  | -1.632 |  | -0.861 |
| (Tan et al. 2023) |  | -0.161 |  | 0.372 |  | -0.432 |  | 0.6659 |  | -0.890 |  | 0.568 |
|  |  |  |  |  |  |  |  |  |  |  |  |  |
| Mean |  | -0.647 |  | 0.256 |  | -2.537 |  | 0.0110 |  | -1.148 |  | -0.146 |

**Note:** Effect sizes are reported as Hedges’ g (negative values indicate lower whole-brain GMV in PSCI compared with controls).**Abbreviations:** GMV, gray matter volume; PSCI, post-stroke cognitive impairment; SE, standard error; CI, confidence interval.

**Supplementary Table 8** Meta-regression analysis of GMV in PSCI on Age

| **Local Maximum** |  |  |  |  |  |  |  | **Cluster** |  |  |  |  |
| --- | --- | --- | --- | --- | --- | --- | --- | --- | --- | --- | --- | --- |
| **Region** |  | **Peak MNI  coordinate  (x, y, z)** |  | **SDM-Z  value** |  | **p value** |  | **No. of voxels** |  | **Local peaks and Cluster breakdown** |  | **(No. of voxels)** |
| **p < 0.05**  **(tfceCorrected)** |  |  |  |  |  |  |  |  |  |  |  |  |
| Right superior frontal gyrus, medial, BA 10 |  | 4,58,12 |  | -2.485 |  | 0.006 |  | 411 |  | Left superior frontal gyrus, medial, BA 10 |  | 148 |
|  |  |  |  |  |  |  |  |  |  | Left superior frontal gyrus, medial orbital, BA 11 |  | 109 |
|  |  |  |  |  |  |  |  |  |  | Left olfactory cortex, BA 11 |  | 41 |
|  |  |  |  |  |  |  |  |  |  | Left superior frontal gyrus, medial, BA 32 |  | 41 |
|  |  |  |  |  |  |  |  |  |  | Left superior frontal gyrus, medial |  | 38 |
|  |  |  |  |  |  |  |  |  |  | Right superior frontal gyrus, medial, BA 32 |  | 8 |
|  |  |  |  |  |  |  |  |  |  | Left anterior cingulate / paracingulate gyri, BA 32 |  | 7 |
|  |  |  |  |  |  |  |  |  |  | Corpus callosum |  | 6 |
|  |  |  |  |  |  |  |  |  |  | Right anterior cingulate / paracingulate gyri, BA 10 |  | 5 |
|  |  |  |  |  |  |  |  |  |  | Right superior frontal gyrus, medial |  | 4 |
|  |  |  |  |  |  |  |  |  |  | Right anterior cingulate / paracingulate gyri |  | 1 |

**Supplementary Table 9** Meta-regression analysis of DTI in PSCI on Age

| **Local Maximum** |  |  |  |  |  |  |  | **Cluster** |  |  |  |  |
| --- | --- | --- | --- | --- | --- | --- | --- | --- | --- | --- | --- | --- |
| **Region** |  | **Peak MNI  coordinate  (x, y, z)** |  | **SDM-Z  value** |  | **p value** |  | **No. of**  **voxels** |  | **Local peaks and Cluster breakdown** |  | **(No. of voxels)** |
| **p < 0.05**  **(tfceCorrected)** |  |  |  |  |  |  |  |  |  |  |  |  |
| Left median network, cingulum |  | -22,-30,-10 |  | -1.956 |  | 0.021 |  | 27 |  | Left median network, cingulum |  | 19 |
|  |  |  |  |  |  |  |  |  |  | Left hippocampus |  | 4 |
|  |  |  |  |  |  |  |  |  |  | Left optic radiations |  | 3 |
|  |  |  |  |  |  |  |  |  |  | Left hippocampus, BA 20 |  | 1 |

**Supplementary Table 10** Meta-regression analysis of DTI in PSCI on MMSE

| **Local Maximum** |  |  |  |  |  |  |  | **Cluster** |  |  |  |  |
| --- | --- | --- | --- | --- | --- | --- | --- | --- | --- | --- | --- | --- |
| **Region** |  | **Peak MNI  coordinate  (x, y, z)** |  | **SDM-Z  value** |  | **p value** |  | **No. of**  **voxels** |  | **Local peaks and Cluster breakdown** |  | **(No. of voxels)** |
| **p < 0.05**  **(unCorrected)** |  |  |  |  |  |  |  |  |  |  |  |  |
| Cerebellum, vermic lobule IV / V |  | -2,-50,-18 |  | -2.952 |  | 0.002 |  | 143 |  | Cerebellum, vermic lobule IV / V |  | 47 |
|  |  |  |  |  |  |  |  |  |  | Left cerebellum, hemispheric lobule IV / V, BA 18 |  | 18 |
|  |  |  |  |  |  |  |  |  |  | Left cerebellum, hemispheric lobule IV / V |  | 14 |
|  |  |  |  |  |  |  |  |  |  | Left cerebellum, hemispheric lobule IV / V, BA 19 |  | 9 |
|  |  |  |  |  |  |  |  |  |  | Left cerebellum, hemispheric lobule III |  | 6 |
|  |  |  |  |  |  |  |  |  |  | Cerebellum, vermic lobule III |  | 5 |
|  |  |  |  |  |  |  |  |  |  | Cerebellum, vermic lobule IV / V, BA 18 |  | 3 |
|  |  |  |  |  |  |  |  |  |  | Left cerebellum, hemispheric lobule IV / V, BA 30 |  | 2 |
|  |  |  |  |  |  |  |  |  |  | Left cerebellum, hemispheric lobule III, BA 30 |  | 1 |
|  |  |  |  |  |  |  |  |  |  | undefined |  | 38 |
| Left arcuate network, posterior segment |  | -46,-42,12 |  | -2.723 |  | 0.003 |  | 62 |  | Left arcuate network, posterior segment |  | 51 |
|  |  |  |  |  |  |  |  |  |  | Left superior longitudinal fasciculus III |  | 5 |
|  |  |  |  |  |  |  |  |  |  | Left middle temporal gyrus, BA 21 |  | 3 |
|  |  |  |  |  |  |  |  |  |  | Left superior temporal gyrus, BA 41 |  | 2 |
|  |  |  |  |  |  |  |  |  |  | undefined |  | 1 |
| Right caudate nucleus |  | 18,22,4 |  | -2.487 |  | 0.006 |  | 58 |  | Right caudate nucleus |  | 28 |
|  |  |  |  |  |  |  |  |  |  | Right anterior thalamic projections |  | 22 |
|  |  |  |  |  |  |  |  |  |  | Corpus callosum |  | 1 |
|  |  |  |  |  |  |  |  |  |  | Right caudate nucleus, BA 47 |  | 1 |
|  |  |  |  |  |  |  |  |  |  | Right caudate nucleus, BA 25 |  | 1 |
|  |  |  |  |  |  |  |  |  |  | Right striatum |  | 1 |
|  |  |  |  |  |  |  |  |  |  | undefined |  | 4 |
| Right middle frontal gyrus, BA 8 |  | 28,12,54 |  | -2.176 |  | 0.015 |  | 50 |  | Right middle frontal gyrus, BA 8 |  | 41 |
|  |  |  |  |  |  |  |  |  |  | Right superior frontal gyrus, dorsolateral, BA 8 |  | 8 |
|  |  |  |  |  |  |  |  |  |  | Right middle frontal gyrus, BA 9 |  | 1 |
| Left fusiform gyrus, BA 20 |  | -30,-28,-28 |  | -2.063 |  | 0.020 |  | 33 |  | Left fusiform gyrus, BA 20 |  | 22 |
|  |  |  |  |  |  |  |  |  |  | Left cerebellum, hemispheric lobule IV / V, BA 20 |  | 4 |
|  |  |  |  |  |  |  |  |  |  | Left fusiform gyrus, BA 30 |  | 3 |
|  |  |  |  |  |  |  |  |  |  | Left cerebellum, hemispheric lobule IV / V |  | 2 |
|  |  |  |  |  |  |  |  |  |  | Left fusiform gyrus |  | 1 |
|  |  |  |  |  |  |  |  |  |  | undefined |  | 1 |
| Corpus callosum |  | -14,-62,54 |  | -2.170 |  | 0.015 |  | 31 |  | Left precuneus, BA 5 |  | 11 |
|  |  |  |  |  |  |  |  |  |  | Corpus callosum |  | 10 |
|  |  |  |  |  |  |  |  |  |  | Left superior parietal gyrus, BA 7 |  | 4 |
|  |  |  |  |  |  |  |  |  |  | Left superior parietal gyrus, BA 5 |  | 3 |
|  |  |  |  |  |  |  |  |  |  | Left precuneus, BA 7 |  | 2 |
|  |  |  |  |  |  |  |  |  |  | Left superior parietal gyrus |  | 1 |
| Right temporal pole, superior temporal gyrus, BA 34 |  | 26,8,-20 |  | -2.095 |  | 0.018 |  | 30 |  | Right temporal pole, superior temporal gyrus, BA 34 |  | 12 |
|  |  |  |  |  |  |  |  |  |  | Right temporal pole, superior temporal gyrus, BA 38 |  | 6 |
|  |  |  |  |  |  |  |  |  |  | Right parahippocampal gyrus, BA 28 |  | 3 |
|  |  |  |  |  |  |  |  |  |  | Right olfactory cortex, BA 48 |  | 2 |
|  |  |  |  |  |  |  |  |  |  | Right amygdala, BA 34 |  | 1 |
|  |  |  |  |  |  |  |  |  |  | Right olfactory cortex |  | 1 |
|  |  |  |  |  |  |  |  |  |  | Right temporal pole, superior temporal gyrus, BA 28 |  | 1 |
|  |  |  |  |  |  |  |  |  |  | undefined |  | 3 |
|  |  |  |  |  |  |  |  |  |  | undefined |  | 1 |
| Left pons |  | -6,-26,-28 |  | -2.50 |  | 0.006 |  | 26 |  | Left pons |  | 18 |
|  |  |  |  |  |  |  |  |  |  | Left cortico-spinal projections |  | 2 |
|  |  |  |  |  |  |  |  |  |  | undefined |  | 5 |
|  |  |  |  |  |  |  |  |  |  | undefined |  | 1 |
| Left temporal pole, middle temporal gyrus, BA 36 |  | -22,6,-40 |  | -1.860 |  | 0.031 |  | 22 |  | Left temporal pole, middle temporal gyrus, BA 36 |  | 11 |
|  |  |  |  |  |  |  |  |  |  | Left fusiform gyrus, BA 36 |  | 11 |
| Right superior frontal gyrus, dorsolateral, BA 9 |  | 22,46,36 |  | -2.009 |  | 0.022 |  | 16 |  | Right superior frontal gyrus, dorsolateral, BA 9 |  | 14 |
|  |  |  |  |  |  |  |  |  |  | Right superior frontal gyrus, dorsolateral |  | 2 |
| Left inferior frontal gyrus, orbital part, BA 47 |  | -46,36,-8 |  | -2.262 |  | 0.012 |  | 15 |  | Left inferior frontal gyrus, orbital part, BA 47 |  | 11 |
|  |  |  |  |  |  |  |  |  |  | Left inferior network, inferior fronto-occipital fasciculus |  | 4 |
| Right superior frontal gyrus, dorsolateral, BA 8 |  | 22,26,50 |  | -2.064 |  | 0.019 |  | 15 |  | Right superior frontal gyrus, dorsolateral, BA 8 |  | 13 |
|  |  |  |  |  |  |  |  |  |  | Right superior frontal gyrus, dorsolateral, BA 9 |  | 2 |
| Left superior frontal gyrus, medial orbital, BA 11 |  | 0,38,-12 |  | -1.966 |  | 0.025 |  | 14 |  | Right superior frontal gyrus, medial orbital, BA 11 |  | 7 |
|  |  |  |  |  |  |  |  |  |  | Left superior frontal gyrus, medial orbital, BA 11 |  | 6 |
|  |  |  |  |  |  |  |  |  |  | Left superior frontal gyrus, medial orbital |  | 1 |
| Right superior frontal gyrus, medial, BA 10 |  | 4,56,20 |  | -2.050 |  | 0.020 |  | 13 |  | Right superior frontal gyrus, medial, BA 10 |  | 6 |
|  |  |  |  |  |  |  |  |  |  | Left superior frontal gyrus, medial, BA 10 |  | 4 |
|  |  |  |  |  |  |  |  |  |  | Left superior frontal gyrus, medial |  | 2 |
|  |  |  |  |  |  |  |  |  |  | Right superior frontal gyrus, medial, BA 32 |  | 1 |
| Left inferior frontal gyrus, orbital part, BA 47 |  | -44,44,-14 |  | -2.401 |  | 0.008 |  | 11 |  | Left inferior frontal gyrus, orbital part, BA 47 |  | 10 |
|  |  |  |  |  |  |  |  |  |  | Left inferior frontal gyrus, orbital part |  | 1 |
| Left fusiform gyrus, BA 37 |  | -30,-44,-20 |  | -1.839 |  | 0.033 |  | 11 |  | Left fusiform gyrus, BA 37 |  | 11 |
| Right frontal aslant tract |  | 48,16,18 |  | -1.885 |  | 0.030 |  | 10 |  | Right frontal aslant tract |  | 6 |
|  |  |  |  |  |  |  |  |  |  | Right inferior frontal gyrus, opercular part, BA 48 |  | 2 |
|  |  |  |  |  |  |  |  |  |  | Right frontal inferior longitudinal fasciculus |  | 1 |
|  |  |  |  |  |  |  |  |  |  | Right superior longitudinal fasciculus III |  | 1 |
